# The Association between Hantavirus Infection and Selenium Deficiency in Mainland China

**DOI:** 10.3390/v7010333

**Published:** 2015-01-20

**Authors:** Li-Qun Fang, Marco Goeijenbier, Shu-Qing Zuo, Li-Ping Wang, Song Liang, Sabra L. Klein, Xin-Lou Li, Kun Liu, Lu Liang, Peng Gong, Gregory E. Glass, Eric van Gorp, Jan H. Richardus, Jia-Qi Ma, Wu-Chun Cao, Sake J. de Vlas

**Affiliations:** 1 State Key Laboratory of Pathogen and Biosecurity, Beijing Institute of Microbiology and Epidemiology, Beijing 100071, China; E-Mails: fang_lq@163.com (L.-Q.F.); zuoshq@126.com (S.-Q.Z.); xinlou2010@163.com (X.-L.L.); liukun5959@163.com (K.L.); 2 Department of Viroscience, Erasmus MC, University Medical Center Rotterdam, Rotterdam 3015CE, The Netherlands; E-Mail: ecmvangorp@gmail.com; 3 Division of Infectious Disease, Chinese Center for Disease Control and Prevention, Beijing 102206, China; E-Mail: wanglp@chinacdc.cn; 4 Environmental and Global Health, College of Public Health and Health Professions, and Emerging Pathogens Institute, University of Florida, Gainesville, FL 32610, USA; E-Mail: ehs.liang@gmail.com; 5 Department of Molecular Microbiology and Immunology, The Johns Hopkins Bloomberg School of Public Health, Baltimore, MD 21205, USA; E-Mails: saklein@jhsph.edu (S.L.K.); ggurrigl@jhsph.edu (G.E.G.); 6 Ministry of Education Key Laboratory for Earth System Modeling, and Center for Earth System Science, Tsinghua University, Beijing 100084, China; E-Mails: liang_lu@tsinghua.edu.cn (L.L.); penggong@mail.tsinghua.edu.cn (P.G.); 7 Department of Public Health, Erasmus MC, University Medical Center Rotterdam, Rotterdam 3000CA, The Netherlands; E-Mails: j.richardus@erasmusmc.nl (J.H.R.); s.devlas@erasmusmc.nl (S.J.V.); 8 National Center for Public Health Surveillance and Information Service, Chinese Center for Disease Control and Prevention, Beijing 102206, China.

**Keywords:** hemorrhagic fever with renal syndrome, selenium, hantavirus, rodents, environmental factors, China

## Abstract

Hemorrhagic fever with renal syndrome (HFRS) caused by hantaviruses and transmitted by rodents is a significant public health problem in China, and occurs more frequently in selenium-deficient regions. To study the role of selenium concentration in HFRS incidence we used a multidisciplinary approach combining ecological analysis with preliminary experimental data. The incidence of HFRS in humans was about six times higher in severe selenium-deficient and double in moderate deficient areas compared to non-deficient areas. This association became statistically stronger after correction for other significant environment-related factors (low elevation, few grasslands, or an abundance of forests) and was independent of geographical scale by separate analyses for different climate regions. A case-control study of HFRS patients admitted to the hospital revealed increased activity and plasma levels of selenium binding proteins while selenium supplementation *in vitro* decreased viral replication in an endothelial cell model after infection with a low multiplicity of infection (MOI). Viral replication with a higher MOI was not affected by selenium supplementation. Our findings indicate that selenium deficiency may contribute to an increased prevalence of hantavirus infections in both humans and rodents. Future studies are needed to further examine the exact mechanism behind this observation before selenium supplementation in deficient areas could be implemented for HFRS prevention.

## 1. Introduction

Hemorrhagic fever with renal syndrome (HFRS) is a zoonotic disease with a severe clinical presentation characterized by, as the name suggests, fever and renal failure potentially complicated by hemorrhage and shock [1]. The disease is the result of infection with an Old-World pathogenic hantavirus, which are negative sensed, single stranded RNA viruses of the genus *Hantavirus*, a member of the *Bunyaviridae* family [2]. Pathogenic hantaviruses are shed in the excreta of their reservoir rodents and in most cases each hantavirus is associated with a specific rodent host [3]. In Mainland China, HFRS is either caused by infection with Hantaan virus (HTNV) or Seoul virus (SEOV) carried by striped field mice (*Apodemus agrarius*) and Norway rats (*Rattus rattus*), respectively [4,5,6,7]. HFRS is a significant public health problem in Mainland China with an annual average incidence reported up to 120 per 100,000 persons for the high endemic parts of the country [7]. Case fatality rates (CFR) range from 0.1% to 15%, depending on the type of hantavirus that caused the infection (SEOVor HNTV) and specific characteristics of the infected individual [8]. To increase insight into HFRS epidemiology in China we previously identified hantavirus counties with the highest HFRS incidence per year in China and defined them as hantavirus “hotspots” [7]. Further analysis of these hotspots revealed that most of them are categorized as being a “selenium-deficient” area. This made us decide to further explore the association between hantavirus infection and selenium deficiency.

Selenium is an essential micronutrient for many life forms, including humans, and has been suggested to play a role in multiple physiological and pathological processes [9,10]. In contrast to many other micronutrients, the intake of selenium varies worldwide, ranging from decreased or even a severe deficient dietary intake to an elevated selenium intake, while even toxic concentrations in crops and feed have been reported in literature [11,12,13]. Low intake of selenium, subsequently leading to decreased selenium levels in the circulation, might directly lead to diseases, such as endemic cardiomyopathy (Keshan disease) or deformative osteoarthritis (Kashin-Beck disease), of which the latter is relatively common in China [14,15,16]. Furthermore, several studies suggested selenium levels influence the occurrence and outcome of multiple other diseases, including both cancer and cardiovascular disease. Most important for this study is the fact that the association between selenium levels and disease outcome is also seen in infectious diseases [17,18]. For instance, the incidence, virulence and progression of viral infections like human immunodeficiency virus (HIV), coxsackie virus, and influenza virus are influenced by selenium deficiency [19,20,21,22]. The exact role of selenium concentration in these infections is not totally understood. However, experimental studies show selenium to play multiple roles in the immune response to infectious diseases. It has been shown that selenoproteins are essential for activated T-cell function and it seems they are involved in the modulation of inflammatory responses, for instance by inducing the interferon response [23,24,25,26]. Selenium deficiency affects T-cell immunity by suppressing T-cell proliferation and causing defects in T-cell-dependent antibody responses [27].

To explore the relation between HFRS incidence and selenium concentration in China, we decided to use a multi-disciplinary translational approach combining ecological, clinical and *in vitro* experiments. The aims of our study are (1) to examine to which extent human HFRS incidence and hantavirus infection rates of rodent reservoirs are associated with measures of selenium deficiency, controlling for other potential confounding environmental factors; and (2) to explore where in the chain of the occurrence of a hantavirus infection, from rodent reservoir to human case, selenium concentration might have its influence. For the second part of this study we decided to study levels of selenium binding proteins in acute HFRS patients (glutathione peroxidase (GPx3)) and the *in vitro* effects of selenium supplementation in a well-established Puumala hantavirus (PUUV) endothelial cell model (HUVEC).

## 2. Results

### Ecological Analysis

**Figure 1:**
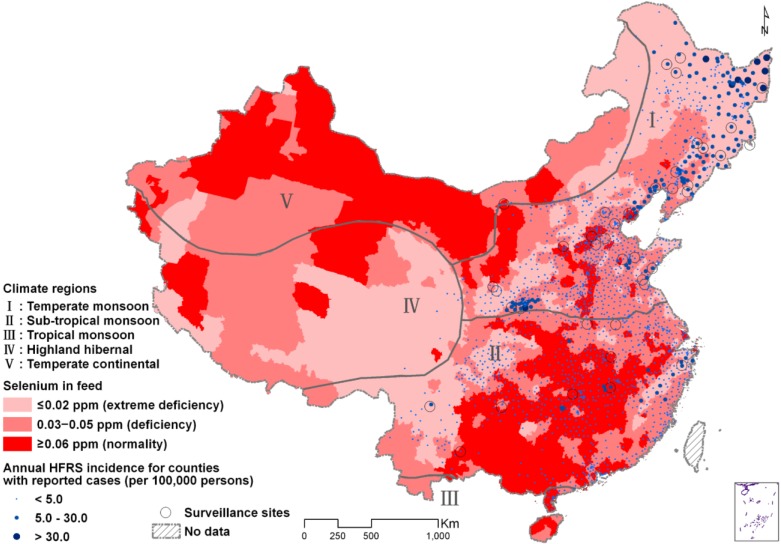
Geographic distribution of hemorrhagic fever with renal syndrome (HFRS) incidence in relation to selenium content of crops and feed in Mainland China. The background of the map with color gradient presents the selenium content of crops and feed, and the dots with size and color gradient display the average annual incidence of HFRS from 2005 to 2010. Areas without dots do not have reported cases of HFRS. The thick grey lines are boundaries of the five climate regions of China. The thin black circles indicate the 40 surveillance sites for hantavirus infection in rodent hosts (used in Figure 2). Ppm is parts per million.

During 2005–2010, a total of 74,118 HFRS cases, including 914 deaths, were reported in 1960 of the 2922 counties in Mainland China. The average annual HFRS incidence varied greatly over counties, ranging from 0 to 54 per 100,000 person-years, with a median of 0.1 per 100,000 person-years. Areas were divided by selenium concentration in crops and feed into selenium non-deficient (≥0.06 parts per million (ppm)), moderate-deficient (0.03–0.05 ppm) and severe-deficient (≤0.02 ppm) areas. The moderate- and severe selenium-deficient areas covered 37.3% and 31.8% of the country, respectively (Figure 1). The average annual HFRS incidence in severe selenium-deficient areas (2.27 per 100,000 person-years) was almost three times higher than in moderate deficient areas (0.83 per 100,000 person-years), which in turn was double that in non-deficient areas (0.40 per 100,000 person-years) (Table 1). Both of the hotspots of highest incidence of HFRS were located on the selenium-deficient belt of China: one in the eastern areas of northeastern China, the other in the central areas of Mainland China (Figure 1).

Univariate Poisson regression analyses revealed that HFRS incidence was significantly associated with selenium content of crops and feed, average elevation, proportion of areas of forests and grasslands, population density, and gross domestic product (GDP). Multivariate analysis showed that the adjusted IRR (7.77) in severe selenium-deficient areas was even higher than the crude IRR (5.68) in comparison with non-deficient areas (Table 1), while elevation, proportion of areas of forests and grasslands, and GDP remained significantly associated with HFRS incidence.

**Table 1:** The association between hemorrhagic fever with renal syndrome (HFRS) incidence and selenium content of crops and feed by Poisson regression, using data from 2005 to 2010 in Mainland China, both univariately and corrected for other influencing factors in multivariate analysis. IRR is incidence rate ratio. CI is confidence interval.

Variables (Unit) ^a^	Average yearly incidence (95% CI, per 100,000 person-years)	Univariate analysis		Multivariate analysis	
		Crude IRR (95% CI)	*p*-value	Adjusted IRR (95% CI)	*p*-value
**Selenium content (categorical, ppm)**					
≥0.06 (non-deficient areas)	0.40 (0.32–0.48)	1	-	1	-
0.03–0.05 (moderate deficient areas)	0.83 (0.70–0.95)	2.07 (1.73–2.46)	<0.001	2.43 (2.04–2.88)	<0.001
≤0.02 (severe deficient areas)	2.27 (1.85–2.70)	5.68 (4.64–6.95)	<0.001	7.77 (6.29–9.61)	<0.001
**Elevation (categorical, 1000 m)**					
<0.4	1.33 (1.14–1.52)	-	-	-	-
0.4–	1.37 (1.05–1.70)	-	-	-	-
0.8–	0.58 (0.35–0.81)	-	-	-	-
1.6–	0.17 (0.00–0.33)	-	-	-	-
>3.2	0.08 (0.00–0.16)	-	-	-	-
**Elevation (continuous, 1000 m)**	-	0.46 (0.41–0.50)	<0.001	0.40 (0.35–0.46)	<0.001
**Croplands (categorical, %)**					
<20	0.98 (0.76–1.20)	1	-	1	-
20–	1.03 (0.84–1.23)	1.04 (0.86–1.26)	0.659	1.14 (0.93–1.39)	0.200
>50	1.13 (0.91–1.35)	1.14 (0.94–1.38)	0.185	1.03 (0.84–1.27)	0.766
**Forests (categorical, %)**					
<5	0.90 (0.72–1.09)	1	-	1	-
5–	1.01 (0.81–1.21)	1.11 (4.62–7.11)	0.285	1.53 (1.26–1.87)	<0.001
>40	1.21 (0.98–1.45)	1.32 (1.42–2.27)	0.004	1.53 (1.26–1.86)	<0.001
**Grasslands (categorical, %)**					
<2	1.20 (1.02–1.38)	1	-	1	-
2–	1.41 (1.13–1.70)	1.18 (0.98–1.41)	0.082	0.92 (0.77–1.10)	0.365
>20	0.44 (0.32–0.55)	0.38 (0.31–0.45)	<0.001	0.58 (0.45–0.74)	<0.001
**Population density (categorical, 1000 persons per km^2^)**					
<0.2	1.38 (1.10–1.65)	-	-	-	-
0.2–	0.98 (0.82–1.14)	-	-	-	-
>0.5	0.73 (0.61–0.84)	-	-	-	-
**Population density (continuous, 1000 persons per km^2^)**	-	0.97 (0.95–0.99)	0.001	1.00 (0.98–1.02)	0.728
**GDP (categorical, 10 million Yuan per km^2^)**					
<0.08	1.22 (0.94–1.50)	-	-	-	-
0.08–	1.07 (0.90–1.25)	-	-	-	-
>0.4	0.84 (0.70–0.96)	-	-	-	-
**GDP (continuous, 10 million Yuan per km^2^)**	-	0.91 (0.88–0.93)	<0.001	0.93 (0.90–0.96)	<0.001

^a^ For all continuous variables, we also report categorical results to allow inspection of the data and to assess whether the continuous assumption was justified. These categorical variables were not considered in the Poisson regression.

Although selenium content was disproportionately spread over the five climate regions of China (Figure 1), similar associations between HFRS incidence and selenium content could be demonstrated within these regions (Table 2). The regions with many severe selenium-deficient areas had the highest IRR in Region I, and in Regions II and III combined. In Region IV, only 58 HFRS cases were reported, but all of them came from severe selenium-deficient areas. Out of the 131 reported HFRS cases in Region V, 28, 103, and 2 cases were located in severe selenium-deficient areas, moderate deficient areas, and non-deficient areas, respectively (Figure 1), and the corresponding annual incidences were 0.073, 0.152, and 0.003 per 100,000 human-years, respectively. Due to the low numbers, statistical analyses were not conducted for Regions IV and V.

**Table 2:** The association between hemorrhagic fever with renal syndrome (HFRS) incidence and selenium content by multivariate Poisson regression in different climate regions, corrected for other influencing factors (not shown). Region I = temperate monsoon climate; Region II = sub-tropical monsoon climate; Region III = tropical monsoon climate. Region II and Region III were combined due to the small area of Region III. In Region IV (highland hibernal climate) and Region V (temperate continental climate), almost all cases were located in severe selenium-deficient and moderate selenium-deficient areas, except for 2 cases located in non-deficient areas in Region V, not allowing statistical analysis due to the low numbers. IRR is incidence rate ratio. CI is confidence interval.

Variables (Unit)	Region I		Region II & Region III	
Selenium content of crops and feed (categorical, ppm)	Adjusted IRR (95% CI)	*p*-value	Adjusted IRR (95% CI)	*p*-value
≥0.06 (non-deficient areas)	1	-	1	-
0.03–0.05 (moderate deficient areas)	1.70 (1.27–2.27)	<0.001	0.85 (0.67–1.08)	0.181
≤0.02 (severe deficient areas)	3.45 (2.49–4.79)	<0.001	2.80 (1.92–4.79)	<0.001

**Figure 2:**
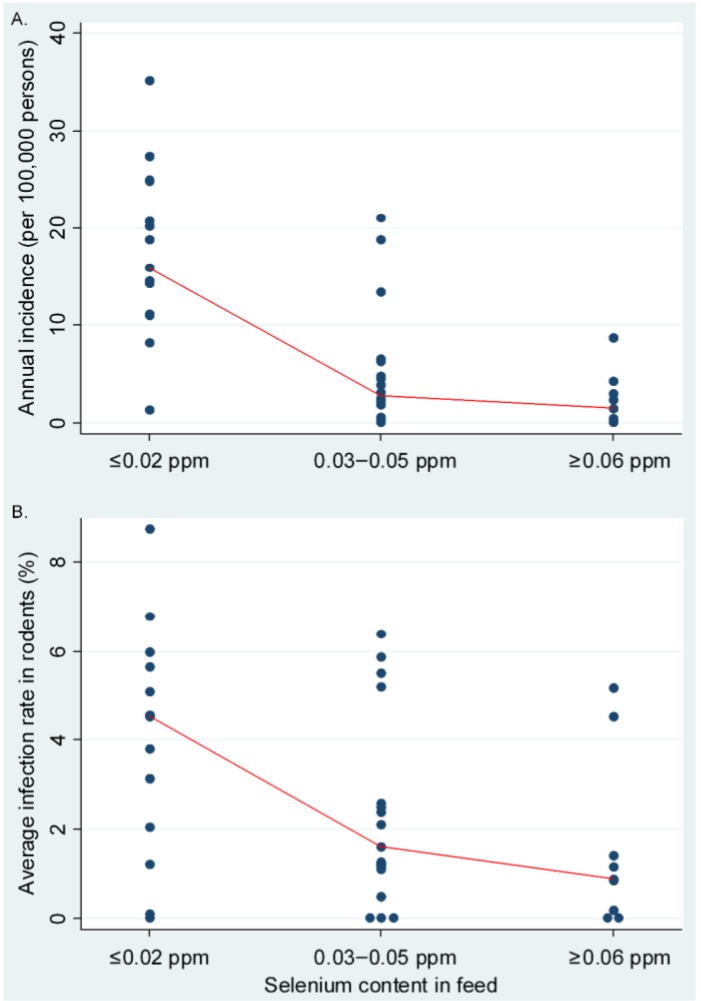
Correlation between both hemorrhagic fever with renal syndrome (HFRS) incidences in humans (**A**) and hantavirus infections in rodents (**B**) and selenium content. The rodent data were from 40 surveillance sites in Mainland China during the years 2005–2008. The points reflect (**A**) average incidences of HFRS in human for the counties each surveillance site and (**B**) annual average hantavirus infections in rodents for each surveillance site, and the lines represent the median bands. Selenium content of crops and feed ≤0.02 ppm represents severe selenium-deficient areas; 0.03–0.05 ppm represents moderate selenium-deficient areas; ≥0.06 ppm represents non-deficient areas.

To verify the association between selenium deficiency in crops and feed and the percentage of infected reservoir rodents, the hantavirus infection rate of rodent hosts in relation to selenium content of crops and feed was investigated for 40 surveillance sites through China (see the circles in Figure 1 for their locations). Both HFRS incidence in humans (Figure 2A) and hantavirus infections in rodents (Figure 2B) were negatively correlated with selenium content of crops and feed. The nonparametric equality-of-medians tests indicated that a significant difference on both HFRS incidence in humans (χ^2^ = 15.6, *p* < 0.001) and hantavirus infection rates in rodents (χ^2^ = 6.8, *p* = 0.034) were found among these three areas.

## 3. Clinical Analysis

### 3.1. Glutathione Peroxidase (GPx3) Concentrations and GPx Activity in Acute HFRS Patients

**Figure 3:**
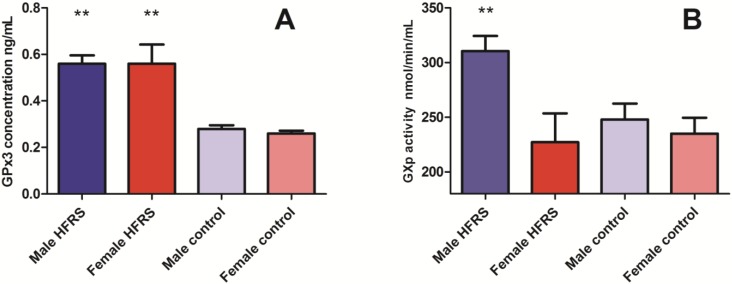
Glutathione peroxidase (GPx3) concentrations (**A**) and GPx activity (**B**) in HFRS patients and healthy controls. Asterisks (**) represent a *p* value smaller than 0.01 (*p* < 0.01). GPx3 levels (**A**), the major binding protein of selenium in humans, were increased in acute HFRS patients compared to controls; Furthermore, GPx enzyme activity levels; (**B**) in nmol/min/mL were statistically significantly higher in male HFRS patients compared to controls.

To study whether selenium levels differed between HFRS cases and healthy controls a case-control study was executed which assessed the glutathione peroxidase 3 (GPx3) concentrations and glutathione peroxidase (GPx) enzyme activity, the major selenium binding proteins in humans [28] in acute HFRS patients and healthy controls. In total, 77 acute HFRS cases were included for GPx3 concentration measurement of which 61 (79.2%) were male. Concentration levels were compared to GPx3 concentrations measured in 72 healthy controls of which 36 were male (50%). Age did not differ significantly between the two groups (*p* = 0.6). GPx3 concentrations in ng/mL are shown in Figure 3. Acute male HFRS patients showed mean GPx3 levels of 0.56 (0.28–0.84 ng/mL), while male healthy controls showed statistically significant lowered GPx3 levels of 0.28 (0.19–0.37) (*p* ≤ 0.001). In female HFRS patients, a similar trend was observed showing statistically significant increased levels of GPx3 in HFRS patients compared to female healthy controls: 0.56 (0.23–0.89 ng/mL) *vs.* 0.26 (0.19–0.33 ng/mL) (*p* = 0.001).

GPx activity levels were measured in a second cohort of HFRS patients. Samples from 80 acute HFRS cases (87.5% male) and 85 healthy controls (54.1% male) were tested for GPx activity. Again, age did not differ significantly between the two groups (*p* = 0.2). Figure 3 visualizes the increased GPx activity levels in acute male HFRS patients *vs.* controls (*p* = 0.001), while there is no significant difference between cases and controls for female HFRS patients.

### 3.2. In Vitro Analysis of the Influence of Selenium on Hantavirus Replication

**Figure 4:**
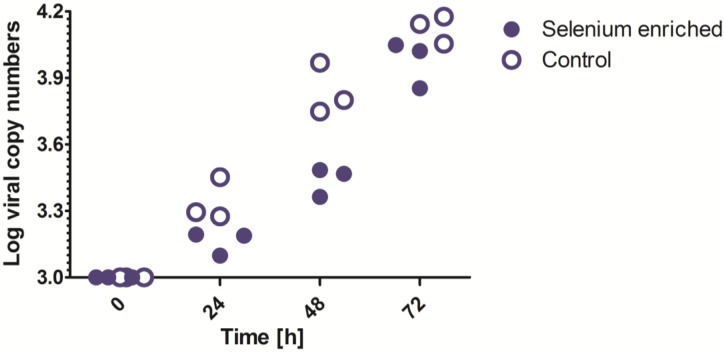
Viral copy numbers with or without the supplementation of selenium to cultured endothelial cells infected with Puumala hantavirus. Selenium supplementation (200 ng/mL of sodium selenite) to the culture medium leads to decreased viral replication after infection with a multiplicity of infection (MOI) of 0.05. Data was 10-log transformed to reach a normal distribution.

To study the influence of selenium concentration on hantavirus replication in a well-established immune competent cell line of human origin we infected Human Umbilical Vein Endothelial Cells (HUVEC) with a low passage isolate of Puumala hantavirus (PUUV) with or without selenium supplementation. Much effort has been put in the isolation of PUUV within four passages over human cell lines to retain its virulence [29] ( Figure 4 shows the viral copy numbers in each replicate measured every 24 h up to 72 h post infection of HUVEC’s with a multiplicity of infection (MOI) of 0.05. Viral copy numbers significantly decreased after selenium supplementation of 200 ng/mL, suggesting a decrease in viral replication. If the number of virus particles of the inoculum increased to MOI 1, or even 3, the decrease in virus replication was no longer significant. This suggests that only at a low percentage of infected cells, after infection with a low MOI, selenium supplementation of 200 ng/mL affects viral replication.

## 4. Discussion

HFRS is one of the most severe viral infectious diseases in Mainland China [6,30]. Furthermore, China is known to be one of the most severely selenium-deficient countries in the world [31,32], while in multiple aspects selenium deficiency is linked to pathophysiological mechanisms [23,25]. By analyzing all reported HFRS cases in Mainland China during a six-year period, we found that the annual HFRS incidence was statistically significantly lower in non-selenium-deficient areas, relative to moderate selenium-deficient and severe selenium-deficient areas.

After adjusting for other influential factors, the incidence of HFRS remained statistically significantly associated with selenium deficiency. In severe selenium-deficient areas, the average HFRS incidence was about six times higher than that in non-deficient areas. The association between HFRS incidence and selenium deficiency was present in all five climate regions of China. Furthermore, HFRS incidence in humans and hantavirus infection rates in rodent hosts were negatively correlated with selenium for 40 surveillance sites across the country. The highly heterogeneous distribution of HFRS incidence and the strong variation in selenium content of crops and feed give us a unique opportunity to explore the possible link between HFRS incidence and selenium.

The low incidence of HFRS in southwestern China, which is a commonly selenium-deficient area, shows that selenium deficiency most likely is not the only determinant of the occurrence of HFRS. A human hantavirus infection is known to be a multi-factorial driven event [33]. Factors influencing reservoir distribution and rodent exposure are known to also play a critical role in the occurrence of HFRS. In our study, we also found low elevation to be an important determinant for HFRS in Mainland China, which may be explained by the preference for low elevation by the HNTV reservoir species, *Apodemus agrarius* [7,34]. Similarly, the distribution of land use (croplands, forests and grasslands), which is associated with agricultural activities, could possibly be related to rodent densities or agricultural rodent exposure. Furthermore, population density and GDP reflect the human living conditions in general and could very well relate to exposure of humans to rodents and their excreta, resulting in increased infections [1]. In southwestern China, the only selenium deficient area without an increase in HFRS incidence, all of the above determinants were unfavorable for HFRS incidence.

The very strong statistically significant association between HFRS incidence and selenium concentration made us further explore which mechanisms potentially underlie this observation. The mechanisms that drive HFRS incidence are complex and multi-factorial [33,35] but basically include three main segments: (1) reservoir ecology; (2) virus ecology; and (3) human factors. Interpretations of earlier studies on selenium and infectious disease suggest a potential role for selenium in each of these segments. By executing the first preliminary experiments on the potential mechanisms between HFRS and selenium, we tried to estimate how selenium concentrations could potentially have their effect. At first we looked at human factors in the relation between selenium and HFRS by performing a case-control study in HFRS cases and comparing the plasma concentration and activity of the major selenium binding proteins GPx and GPx-3 [36]. We thereby hypothesized that deficiency in selenium could either increase the hosts’ susceptibility to hantavirus infection, leading to more infections, or increase the host response to the hantavirus infection, which would lead to a more severe disease and thus an increase of reported HFRS cases. However, HFRS patients showed an increase in GPx3 levels, and also GPx activity was significantly higher in acute HFRS male patients. This observed pattern seems contradictory to the hypothesis that selenium deficiency affects the human factor in the occurrence of a HFRS case, especially since other studies describe a decrease in GPx3 activity during viral infection and sepsis [28,37], and suggests that the possible mechanism is more likely to have its effect on the reservoir rodent or the virus.

Our preliminary *in vitro* data show that selenium supplementation decrease viral replication in immune competent cells after infection with a (very) low infectious dose. However, increasing the infectious dose eliminated this effect, suggesting a role for selenium only in low viral load infections *in vitro* and thereby a (weak) immune stimulant effect of selenium supplementation. This effect could be bigger *in vivo* since earlier observed effects of selenium supplementation on natural killer- and T cell activity do not play a role in the *in vitro* cell model [27]. We therefore hypothesize that the mechanism behind our observations does not lie in the human susceptibility or response to infection but most likely concerns a prolonged or increased viral load in the reservoir rodent. The effect of selenium depletion on the rodents could potentially lead to prolonged and increased virus shedding as a result of a selenium dependent decrease in immunity. Furthermore, a direct mutating effect of selenium on the virus, resulting in changes in the viral fitness, cannot be excluded as well. This observation that has also been described in influenza virus research [38,39]. To study this possible effect in HNTV and SEOV would warrant the use of low passage cultured virus stocks, preferably isolated on rodent cell lines, since it has been proven that hantavirus strains rapidly adapt to passages over human cell lines [40,41]. Unfortunately, with our current assays and samples available, we were not able to measure selenium concentrations in the reservoir rodents. To fully address these questions, future experiments should focus on selenium levels in reservoir species. Animal experiments with selenium supplements and depletion and their relation to immune status and viral shedding have the highest priority.

In this study, a statistically significant ecological association between HFRS incidence and selenium deficiency was found, also after adjusting for other environmental and social-demographic variables. However, the lack of more precise data on the selenium content of crops and feed and additional data on selenium contents in soils, food grains, and the human body is a limitation of the study. A further drawback of our study is that our preliminary experiments exploring a potential mechanism behind this observation did not result in a clear conclusion. Still, our results provide possible targets for focused prevention and control of HFRS (*i.e.*, the areas of selenium deficiency with low elevation, few grasslands, or an abundance of forests). Additional experimental studies should further explore possible mechanisms of how selenium deficiency leads to an increased risk of HFRS. If this relationship holds, then this would provide rationale for preventive measures of HFRS, such as providing selenium supplements, either through fertilization of crops or as a preventive medicine for humans in severe selenium-deficient regions.

## 5. Materials and Methods

### 5.1. Epidemiological Data Collection and Management

The acquirement and use of human data was approved by the Ethical Review Board, Science and Technology Supervisory Committee at the Beijing Institute of Microbiology and Epidemiology. Since 1950, HFRS has been included in the list of notifiable infectious diseases in Mainland China. Cases were reported using a standard protocol formulated by the Chinese Center for Disease Control and Prevention (CISDCP). HFRS was initially diagnosed clinically by signs and symptoms according to the guideline issued by the Ministry of Health, China. Since 1982, reported cases have also been confirmed by standard serological tests, such as enzyme-linked immunosorbent assays and indirect immuno-fluorescence assays [6,42]. In this study, we included all HFRS cases reported to the CISDCP during 2005 to 2010. Information on age, sex, occupation, residence address, working address, onset date and location, hospital admission date, and clinical outcomes were retrieved from the diagnostic application form sent to the reference laboratory. Demographic data of each county were obtained from the National Bureau of Statistics of China (Beijing, China).

Information on hantavirus infections in rodent hosts was collected from 40 surveillance sites across the country according to a protocol developed by CISDCP [43]. Several investigation spots were selected within each surveillance site for surveys of rodent hosts twice every year: March–April and September–October. A total of 100–150 traps per patch with peanuts as bait were placed for 2–3 consecutive nights at each investigation spot and at least 200 rodents were caught from each surveillance site every year. Lung tissues of rodents were collected and stored in liquid nitrogen, and then examined for hantavirus antigens using indirect immunofluorescent assay (IFA), as previously described by Lee and others [44].

To assess the association between the number of HFRS cases and selenium deficiency, the investigation data on selenium content of crops and animal feed in various areas were obtained from the Chinese Academy of Agricultural Sciences [45]. All counties were classified into 3 categories according to the average selenium concentration of crops, including corn, barley, rice, sorghum, millet, potatoes, broad beans, wheat, *etc.*, grown in that areas: non-selenium-deficient areas (≥0.06 ppm), moderate selenium-deficient areas (0.03–0.05 ppm), and severe selenium-deficient areas (≤0.02 ppm) [45]. Data on geographic and social-demographic co-variables, possibly associated to the incidence of HFRS, including elevation, land use (*i.e.*, distribution of croplands, forests and grasslands), population density, and gross domestic product (GDP), were also collected. Elevation data were derived from a shuttle radar topography mission database with a spatial resolution of 1 km [46]. Data of land use and GDP were collected with a spatial resolution of 1 km from the Institute of Geographical Sciences and Natural Resources Research, Chinese Academy of Sciences. The population density of each county was obtained from the National Bureau of Statistics of China. The average elevation, proportion of area of land use, population density, and average GDP for each county were then extracted by overlapping the county boundary and each of co-variables using ArcGIS 9.2 software (ArcGIS 9.2, Environmental Systems Research Institute, Redlands, CA, USA).

### 5.2. Spatial Analysis of HFRS Incidence

Each HFRS case was geo-referenced to the corresponding polygons of the China digital map through the linkage of the 6-digit county geo-code. The average annual incidence was calculated for each county, and a thematic map was created and overlapped onto the map of selenium concentration of crops and feed by using ArcGIS 9.2 (ESRI Inc., Redlands, CA, USA, 2010). Based on average annual incidence, all counties were grouped into four HFRS categories: non-endemic, low endemic (incidence <5.0 per 100,000 person-years), medium endemic (5.0 to 30.0 per 100,000 person-years), and high endemic (>30.0 per 100,000 person-years).

### 5.3. Ecological Analysis

We used a Poisson regression to test for a relationship between incidence of HFRS and selenium deficiency. The cumulative number of HFRS cases per county was set as the outcome variable, and population size of each county was included as offset. The environmental and social-demographic factors, average elevation, proportion of areas of croplands, forests, and grasslands, population density, and average GDP for each county, were included as co-variables. For each continuous variable, we also reported the average yearly incidence of HFRS for different categories to allow inspection of the data and to assess whether the continuous assumption was justified. The categorical results for continuous variables were not included in the Poisson regression. The incidence rate ratio (IRR) in response to the change of the variable by a given amount (1000 m for elevation, 1000 persons per km^2^ for population density, and 10 million Yuan per km^2^) was used to determine the impact of each variable on HFRS incidence. The 95% confidence interval (CI) and corresponding P-value were estimated after correcting for over dispersion, because of the nature of infectious diseases with spatial clustering patterns [44]. First, univariate analyses were performed to examine the effect of each variable separately, and then multivariate analysis was performed by including all co-variables with a *p* < 0.20 in the univariate analysis. We also performed the regression analyses separately for the 5 climate regions: temperate monsoon (I), sub-tropical monsoon (II), tropical monsoon (III), highland hibernal (IV), and temperate continental (V) [47].

To verify the influence of selenium deficiency on hantavirus infection in rodents, we investigated the association between selenium content of crop and animal feed in each county and hantaviruses infection rates in the rodent hosts caught from the 40 surveillance sites in the period from 2005 to 2008. Hantavirus infection rates were plotted against the selenium content category (non-deficient areas, moderate deficient areas and severe deficient areas). The differences in infection rates were examined using the nonparametric equality-of-medians test [48].

### 5.4. Clinical Epidemiological Study

#### Selenium Levels in Acute HFRS Patients and Controls

To study whether the occurrence of a HFRS case is associated with lowered circulatory selenium levels, we compared HFRS cases to non-infected healthy controls. We decided to measure the plasma activity and concentration of one of the major selenium binding proteins in human serum: glutathione peroxidase (activity of collective GPx and concentration of glutathione peroxidase-3 (GPx-3). Patient samples were used anonymously after written informed consent. GPx activity and GPx-3 concentration in the circulation is directly correlated to selenium concentrations [28]. Blood samples of HFRS patients were collected, after informed consent, from spring 2009 to winter 2010 at the Xi’an No.8 Hospital in the Shaanxi Province, China. All cases showed conspicuous clinical signs of HFRS and whose blood samples were tested by laboratory experiment (HNTV RT-PCR positive, or IgM, or IgG HNTVG-antibody positive). Eighty-five control (non-HFRS patients and negative hantavirus serology) samples were collected among healthy volunteers in Xi’an, Shaanxi Province. All plasma samples were stored in −80 °C until use.

GPx activity was measured by commercially available Glutathione Peroxidase Assay Kit (BioVision, Milpitas, CA, USA) through a coupled reaction with glutathione reductase (GR) according to the manufacturers manual. Briefly, in the assay, GPx reduce Cumene Hydroperoxide, and oxidize glutathione (GSH) to glutathione (GSSG). The generated GSSG is reduced to GSH with consumption of NADPH by GR. The decrease of NADPH is proportionally to GPx activity in the reactions. The decrease of NADPH was measured by absorbance at 340 nm, which was used to calculate the GPx Activity.

GPx3 concentration was measured using an Enzyme-linked Immunosorbent Assay Kit as by manufacturer’s instructions (Uscn Inc. Wuhan, China). Plasma samples were prepared in two dilutions and each dilution was measured in duplicate. Briefly, standards or plasma samples were added to the microtiter plate with pre-coated GPx3 specific antibody. The plates were washed after they were incubated at 37 °C for 2 h. After biotinylated GPx3 antibody were added and incubated at 37 °C for 1 h, the plates were washed again. Horseradish Peroxidase (HRP) conjugated Advin was added and incubated for 30 min. After the plates were washed, TMB substrate solution was added. The reaction was terminated by addition of sulfuric acid solution. The samples’ absorbance was read at 450 nm using a plate reader. The GPx3 concentration was calculated by the standard curve.

### 5.5. *In Vitro* Assessment of the Influence of Selenium on Hantavirus Replication

To study the influence of selenium concentration on hantavirus replication we studied the kinetics of the infection of an immune competent cell line (primary isolated human umbilical vein endothelial cells (HUVEC)) with a low passage hantavirus isolate. This model was chosen based on the relation between immune activation (*i.e.*, interferon inducement) and selenium concentrations, mentioned in the introduction [25,27,28]. HUVEC were harvested from fresh umbilical veins, kindly provided by the Erasmus MC birth center. Cells were isolated as described in [29]. Briefly, Human umbilical vein endothelial cells (HUVEC) were harvested from umbilical veins (kindly provided by the Erasmus MC birth-center, Rotterdam, the Netherlands). Umbilical cords were stored in sterile 500 mL PBS + gentamycin (50 µg/mL) (Leo Pharmaceutical Products, Ballerup, Denmark). The veins were rinsed with PBS containing 50 U/mL heparin (Leo Pharmaceutical Products). Subsequently, cells were detached with 0.1% collagenase solution (C6885, Sigma Aldrich, St. Louis, MO, USA). Cell suspension was collected in a sterile 50 mL tube followed by two times centrifugation (5 min; 300× *g*). The cell pellet was re-suspended in HUVEC medium (human endothelial-SFM medium) (Invitrogen, Life Technologies, Thermo Fisher Scientific Carlsbad, CA, USA) containing 10% human serum (Lonza, Breda, The Netherlands), 20% filtrated FBS; penicilin/streptomycin 100 U/mL, 20 ng/mL fibroblast growth factor (Peprotech, Rocky Hill, NJ, USA) and 10 ng/mL of endothelial cell growth factor (Peprotech). Cell suspensions were cultured in flasks pre-coated with 20 μg/mL of fibronectin (Roche, Woerden, The Netherlands). Passage two cells from one specific donor were used for this study. The identity of the endothelial cells was confirmed by flow cytometry using *Ulex europeus* lectin (EY laboratories, San Mateo, CA, USA), anti-CD31 antibody (Sigma Aldrich, St. Louis, MO, USA) and Von Willebrand Factor staining (Dako, Heverlee, Belgium). HUVEC were seeded into 24-well plates (Corning, St. Harrodsburg, KY, USA). Confluent monolayers were infected with a multiplicity of infection (MOI) of 0.05, 1 or 3, with infectious Puumala (PUUV) hantavirus freshly isolated from chronically infected bank voles (*Myodes glareolus*), kindly provided by Heikki Henttonen, METLA Forest Research Institute, Vantaa, Finland. Cells were incubated for 60 min at 37 °C in 5% CO_2_. After incubation, the supernatant was discarded and cells were washed three times with RPMI 1640 (Gibco, Life Sciences, Waltham, MA, USA). Subsequently, 500ul of fresh HUVEC medium was added to the wells without addition of selenium or supplemented with 200 ng/mL of cell culture suitable sodium selenium (Sigma Aldrich). HUVEC were incubated for 72 h at 37 °C in 5% CO_2_. Viral replication was determined by RNA copy number quantification as described [49]. The quantity of viral RNA was measured with in a real-time qRT-PCR assay (TaqMan^®^Fast Virus 1-Step Master Mix, Invitrogen, Life Technologies, Thermo Fisher Scientific, Carlsbad, CA, USA) using Applied Biosystems^®^ 7500 Real-Time PCR system (Life Technologies, Thermo Fisher Scientific, Waltham, MA, USA). The RNA copy number in each sample was calculated from a standard RNA curve generated by an *in vitro* transcribed PUUV RNA standard derived from *in vitro* RNA transcripts generated using a segment amplified with pan-hantavirus degenerative PCR primers as described [50].
